# Cold Priming Induced Tolerance to Subsequent Low Temperature Stress is Enhanced by Melatonin Application during Recovery in Wheat

**DOI:** 10.3390/molecules23051091

**Published:** 2018-05-04

**Authors:** Luying Sun, Xiangnan Li, Zongshuai Wang, Zhongwei Sun, Xiancan Zhu, Shengqun Liu, Fengbin Song, Fulai Liu, Yongjun Wang

**Affiliations:** 1 Key Laboratory of Mollisols Agroecology, Northeast Institute of Geography and Agroecology, Chinese Academy of Sciences, Changchun 130102, China; sunluy1@126.com (L.S.); zhuxiancan@iga.ac.cn (X.Z.); lsq@iga.ac.cn (S.L.); songfb@iga.ac.cn (F.S.); 2 Crop Research Institute, Shandong Academy of Agricultural Sciences, Jinan 250100, China; wzshuai0109@163.com; 3 Agricultural Research Institute of Xu-huai Region, Lianyungang Academy of Agricultural Science, Lianyungang 222000, China; njausunzw@163.com; 4 Faculty of Science, Department of Plant and Environmental Sciences, University of Copenhagen, Højbakkegård Allé 13, DK-2630 Tåstrup, Denmark; fl@plen.ku.dk; 5 Institute of Agricultural Resources and Environment, Jilin Academy of Agriculture Sciences/State Engineering Laboratory of Maize, Changchun 130033, China

**Keywords:** cold tolerance, melatonin, *Triticum aestivum*, mineral nutrition, priming

## Abstract

Cold priming can alleviate the effects of subsequent cold stress on wheat plant growth. Melatonin plays a key role in cold stress response in plants. In this study, the effects of foliar melatonin application during recovery on the cold tolerance of cold primed wheat plants were investigated. It was found that both melatonin and cold priming increased the photosynthetic rate and stomatal conductance, enhanced the activities of antioxidant enzymes, and altered the related gene expressions in wheat under cold stress. Melatonin application is helpful for the photosynthetic carbon assimilation and membrane stability of the cold primed plants under cold stress. These results suggested that foliar melatonin application during recovery enhanced the cold priming induced tolerance to subsequent low temperature stress in wheat.

## 1. Introduction

Low temperature stress is one of the critical abiotic stress limiting the plant growth and accounting for the considerable grain yield loss in wheat (*Triticum aestivum* L.) [1]. The common low temperature stress effects on wheat plant include overproduction of reactive oxygen species (ROS) [2], reduction of photosynthetic carbon assimilation [3], depression in the activity of antioxidant enzymes [4], rigidification of membranes [5], and changes in gene expression [6]. Low temperature thermodynamically depresses the kinetics of metabolic reactions in plants tissues [7]. It also causes thermodynamic disequilibrium, which directly affects the stability and the solubility of the globular proteins [8]. This further results in the inactivity of many metabolic enzymes and disturbance of the metabolic regulations. In addition, low temperatures regulate the formation of RNA secondary structures and then affects the related gene expressions [9].

Higher plants have developed strategies to adapt to low temperature stress and thus, obtain stress tolerance [10]. In transcriptome level, cold stress induces differential expression of many genes, such as *VRN1*, *C-repeat Binding Factor* (*CBF*), *COLD-ACCLIMATION SPECIFIC30* (*CAS30*), which are required for obtaining the ability to tolerate freezing conditions [11]. Various phytohormones and chemicals were reported to enhance cold tolerance in various plant species, such as nitric oxide and gibberellin [1]. Cold priming has been reported to have stronger impacts on plant fitness than cold acclimation in spring in *Arabidopsis thaliana* [12]. Winter wheat originates from temperate regions and can possess tolerance to low temperature stress [13]. Our previous study has documented that early exposure to a moderately sub-optimal chilling temperature can prepare the wheat more quickly and actively to respond to subsequent cold stress [14]. This process is referred to cold priming, which is involved in many physiological responses. For example, in wheat plants, the oxygen scavenging systems activated by cold priming play an important role in promoting cold tolerance [3,14]. The cold priming activates the antioxidant systems in chloroplasts and mitochondria, depressing the oxidative burst in photosynthetic apparatus, thus enhancing the tolerance to subsequent low temperature stress in wheat [14].

As a molecule with various functions in plant physiological response, melatonin (*N*-acetyl-5-methoxytryptamine) is proven to play a key role in regulating stress tolerance in many plant species such as wheat [15,16,17,18,19,20]. For example, melatonin application significantly enhances the drought tolerance of plants by regulating the mitochondria synthesize melatonin levels [19]. It was currently documented that melatonin application could alleviate cold-induced adverse effects in tea plants by reducing oxidative stress through enhanced antioxidant potential and redox homeostasis [21]. In rice, low doses of melatonin application could also relieve the cold stress-induced inhibitions to photosynthesis and photosystem II activities via increasing antioxidant enzyme activities and non-enzymatic antioxidant levels [22]. Our previous study showed that melatonin application at grain filling in maternal wheat plants promoted the cold tolerance of wheat offspring at the seedling stage through accelerating the starch degradation in germinating seeds, activating the antioxidant enzymes, and enhancing the photosynthetic electron transport efficiency [15]. However, the roles of melatonin in cold priming induced cold tolerance in wheat is rarely known.

In the present study, the single and combined effects of cold priming and melatonin application during recovery stage after cold priming on the photosynthetic capacity and electron transport system efficiency, antioxidant enzyme system, and related gene expressions were investigated. Our hypotheses were that: (1) melatonin benefits the plant growth under cold stress by regulating the photosynthetic electron transport system and antioxidant enzyme system in cold primed wheat plants; (2) melatonin application enhances the cold priming induced tolerance to subsequent cold stress in wheat.

## 2. Results

**Figure 1:**
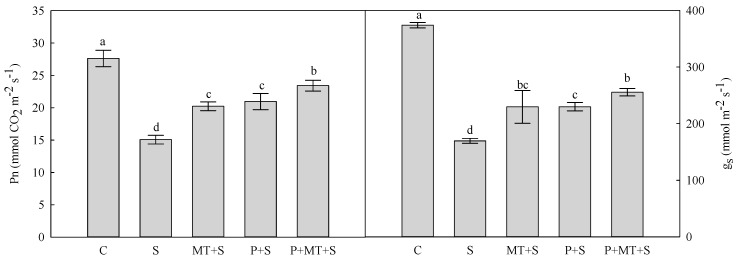
Net photosynthetic rate (*Pn*) and stomatal conductance (*g_s_*) of top leaf in wheat as affected by cold priming, melatonin treatment, and subsequent cold stress. N, non-stress control; S, low temperature stress; MT+S, foliar melatonin treatment + low temperature stress; P+S, cold priming + low temperature stress; P+MT+S, cold priming + foliar melatonin treatment + low temperature stress. The foliar treatment with melatonin during recovery is indicated by green arrows.

As expected, 3-day low temperature stress (2/0 °C in the day/night) had a large effect on photosynthetic rate (*Pn*) and stomatal conductance (*g_s_*) in wheat. Compared to the control plants (C), the *Pn* and *g_s_* were decreased by 45% and 55% in wheat plants under cold stress (S), respectively (Figure 1). The reductions of *Pn* and *g_s_* were significantly alleviated by single and combined treatment of cold priming and foliar melatonin application. In addition, the plants under P+MT+S showed the highest *Pn* and *g_s_* under cold stress.

**Figure 2:**
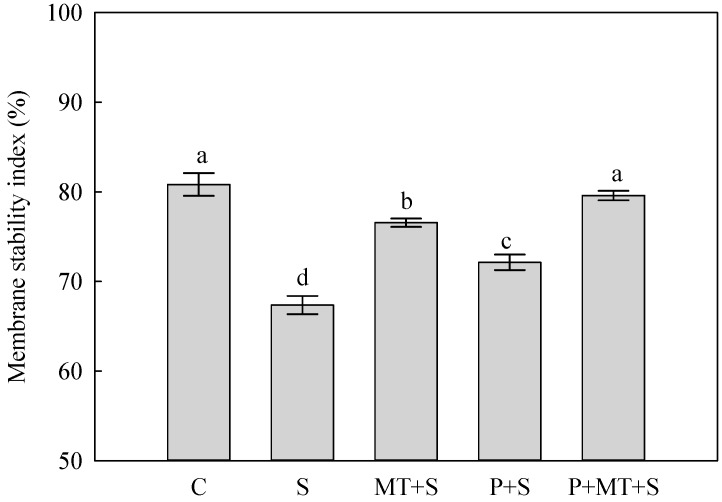
Membrane stability index (MSI) of top leaf in wheat as affected by cold priming, melatonin treatment, and subsequent cold stress. Abbreviations of treatments are explained in Figure 1.

**Figure 3:**
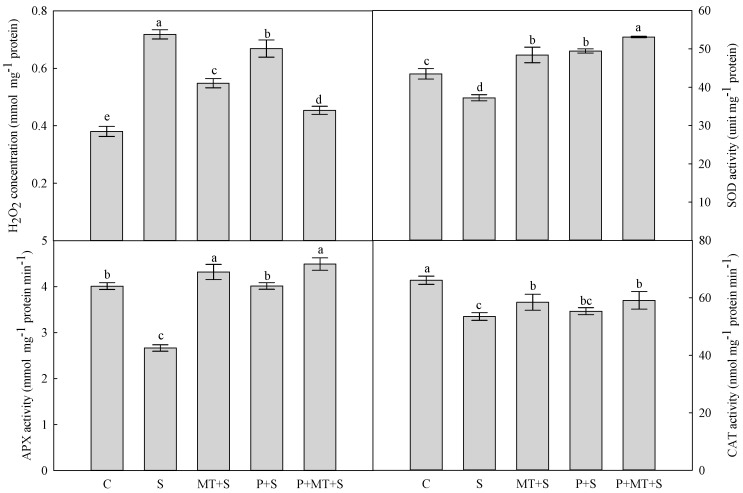
Concentration of H_2_O_2_ and activities of superoxide dismutase (SOD), ascorbate peroxidase (APX) and catalase (CAT) in top leaf of wheat as affected by cold priming, melatonin treatment, and subsequent cold stress. Abbreviations of treatments are explained in Figure 1.

The cold stress significantly depressed the membrane stability index (MSI) of the top leaf in wheat plants in comparison to the control (Figure 2). When exposed to the subsequent cold stress, the highest MSI was found in P+MT+S plants, followed by MT+S plants and P+S plants, and the S plants had the lowest level. The cold stress significantly increased the H_2_O_2_ concentration in wheat leaves, compared with the non-stress control (Figure 3). Though the plants under MT+S, P+S and P+MT+S all had significantly higher H_2_O_2_ concentration than C plants, the increase in leaf H_2_O_2_ concentration was the lowest in P+MT+S plants among these treatments.

**Figure 4:**
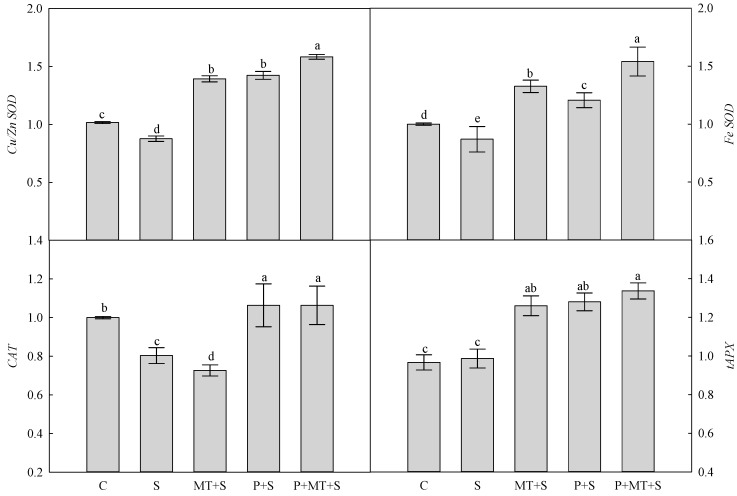
Relative transcript abundance of *Cu/Zn SOD*, *Fe SOD*, *CAT*, and *thylakoid-bound APX (tAPX)* in the top leaf of wheat as affected by cold priming, melatonin treatment, and subsequent cold stress. Abbreviations of treatments are explained in Figure 1.

The cold stress decreased the superoxide dismutase (SOD) activity in wheat leaves, compared with the control (Figure 3). The single/combined treatments of cold priming and melatonin application all enhanced the SOD activity in wheat leaves, and the highest SOD activity was observed in the plants under P+MT+S. In addition, the activities of APX and CAT were both reduced by the cold stress, compared with the non-stress control. The activity of APX was significantly enhanced by cold priming, melatonin application, and their combination, while the activity of CAT was only slightly affected by these treatments. Compared with the control, the expression of *Cu/Zn SOD* gene was reduced by cold stress, while it was significantly increased by the single and combined treatment of cold priming and foliar melatonin application (Figure 4). A similar trend was found in the expression of *Fe SOD* gene. The expression of *CAT* gene was significantly reduced by S and MT+S, compared with C; however, it was increased by P+S and P+MT+S, though it was not statistically significant. No significant difference was found in the expression of *tAPX* between C and S. The expression of *tAPX* was enhanced by MT+S, P+S and P+MT+S, compared with the control.

**Figure 5:**
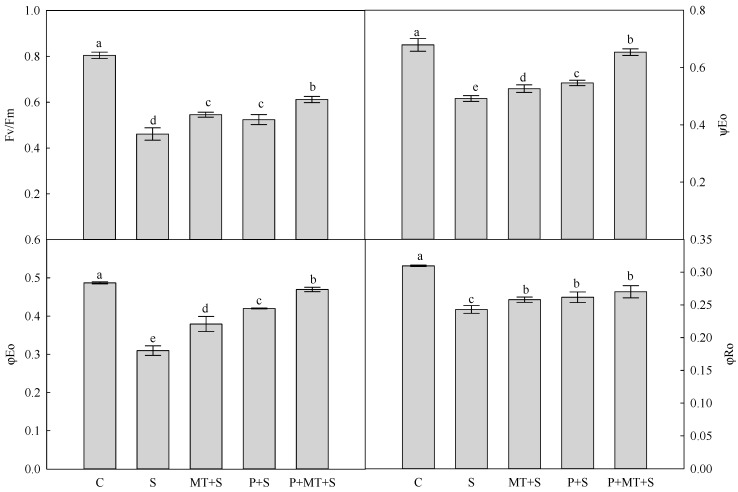
Fluorescence transient chlorophyll *a* parameters deduced from analysis of the JIP-test of wheat leaf as affected by cold priming, melatonin treatment, and subsequent cold stress. Fv/Fm, maximum quantum yield of Photosystem II (PSII); PI_ABS_, performance index on absorption basis; φ_PO_, Maximum quantum yield for primary photochemistry; φ_EO_, Quantum yield for electron transport (ET); φ_RO_, Quantum yield for reduction of end electron acceptors at the PSI acceptor side (RE); ψ_EO_, Probability that an electron moves further than Q_A_; Abbreviations of treatments are explained in Figure 1.

The maximum quantum efficiency of photosystem II (Fv/Fm) was reduced by 43% in the wheat leaves under cold stress compared with the control (Figure 5). The highest Fv/Fm was found in the combined cold priming and melatonin treatment when exposed to subsequent cold stress. A similar trend was observed for ψEo and φEo, which represents the probability that an electron moves further than Q_A_ and quantum yield for electron transport (ET), respectively. In comparison to the control, the quantum yield for reduction of end electron acceptors at the PSI acceptor side (RE) (φRo) was depressed by cold stress. Under cold stress, the φRo was significantly higher in MT+S, P+S and P+MT+S, when compared with S. In addition, there was no significant difference in φRo among MT+S, P+S and P+MT+S under cold stress.

**Figure 6:**
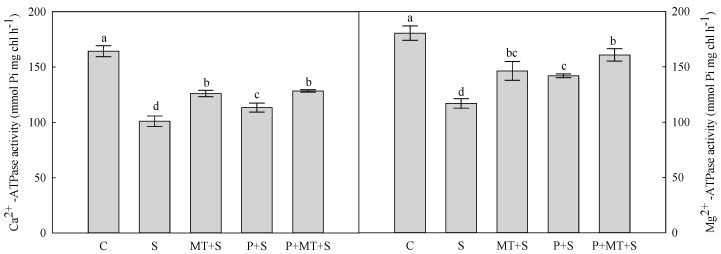
Activities of Ca^2+^-ATPase and Mg^2+^-ATPase in top leaf of wheat as affected by cold priming, melatonin treatment, and subsequent cold stress. Abbreviations of treatments are explained in Figure 1.

The activities of Ca^2+^-ATPase and Mg^2+^-ATPase are important for the ATP formation, which were both reduced by 3-day cold stress (Figure 6). When exposed to the subsequent cold stress, MT+S and P+MT+S plants possessed higher Ca^2+^-ATPase than P+S plants. However, the P+MT+S plants had higher Mg^2+^-ATPase than MT+S and P+S plants under cold stress.

## 3. Discussion

Both *Pn* and *g_s_* of top leaf in wheat were remarkably reduced by 3-day cold stress (2/0 °C in the day/night), compared with the non-cold control. Our previous semi-field study showed that 7-day cold priming (5.2 °C lower temperature than the ambient temperature) at the Zadoks growth stage 28 resulted in higher *Pn* during the subsequent low temperature stress in wheat [14]. In the present pot experiment, a similar result was found in which P+S plants had higher *Pn* and *g_s_* than S plants. Furthermore, the foliar melatonin application during the recovery after cold priming significantly enhanced the *Pn* of wheat plants, indicating that the foliar melatonin application benefited the increase in *Pn* of cold primed plants under subsequent cold stress. This could be partly explained by the higher *g_s_* of top leaf in wheat [23,24].

Several cold stress tolerance mechanisms were related to improved membrane stability, which enabled the wheat plants to avoid the low temperature induced damage [25]. Here, it was observed the membrane stability in wheat leaves was enhanced by both cold priming and foliar melatonin application, and when both of them were applied, the MSI was as high as that of under the non-stress control. This illuminated that combination of cold priming and foliar melatonin application during recovery could mitigate the injury of cold stress to the membrane in wheat leaves. In many plant species, when exposed to abiotic stress, the higher membrane stability was closely related to the upregulated ROS scavenging processes, such as enhanced antioxidant enzyme activity and increased level of non-enzymatic antioxidants [26,27]. In the present study, the leaf H_2_O_2_ concentration was significantly reduced by cold priming and melatonin treatment, compared with S treatment. In the antioxidant system, SOD catalyzes the disproportionation of singlet oxygen to produce H_2_O_2_. After this process, H_2_O_2_ is decomposed to H_2_O and O_2_ with the combination of CAT and APX, which was called “water to water cycle” [28,29]. Here, the SOD and APX activities were enhanced by cold priming, melatonin treatment, and their combination, while the CAT activity was not significantly affected by these treatments. In addition, it should be noted that the highest activities of SOD and APX were found in the combined treatment of cold priming and melatonin application. This indicated that the depressed H_2_O_2_ level under cold stress was mainly due to the activated SOD and APX in combined cold priming and melatonin treated wheat plants. In our previous study, it was found the SOD, APX and CAT were all involved in the regulation of melatonin in the drought priming induced cold tolerance in barley [30]. Under cold stress, the expressions of *Cu/Zn SOD* and *Fe SOD* were significantly upregulated in cold primed plants and melatonin treated plants. In consistent with the dynamics of antioxidant enzyme activity, the *CAT* expression was not significantly affected by P+S and P+MT+S; whereas, it was reduced by MT+S. All these results indicated that CAT perhaps did not play an important role in the regulation of melatonin in the cold priming induced cold tolerance in wheat. In addition, the *tAPX* expression was significantly upregulated by cold priming, melatonin treatment, and their combination in wheat, which was in line with the changes of APX activity. It suggested that melatonin application might affect the endogenous melatonin level, which further regulated the antioxidant system for a better ROS balance.

Photosynthesis converts light energy to ATP and redox equivalents, which is important to support the plant growth [31,32,33,34]. Light energy is trapped by the antenna of photosystem to drive the charge separation in the reaction centers [34]. This process is easily disturbed by cold stress, which could be monitored with chlorophyll *a* fluorescence analysis [3,35,36]. In this study, the foliar melatonin application during recovery enhanced Fv/Fm in the cold primed wheat plants, indicating that melatonin could increase the quantum yield of PS II and donation to PS II in cold primed wheat under cold stress [37]. The efficiency in balancing the dark reactions after Q_A_, expressed by ψEo, was also increased by the combination of cold priming and melatonin application under subsequent cold stress. This indicated that the melatonin enhanced the cold primed plant’s efficiency in the process that trapped exciton transfers electron into the electron transport chain beyond Q_A_ [38]. The maximum quantum yield of primary photochemistry and quantum yield for the reduction of the end electron acceptors at PS I acceptor side, which was indicated by φEo and φRo respectively, were both enhanced by cold priming and melatonin treatment. This showed that melatonin induced higher φEo and φRo that are helpful for photosynthetic electron transport efficiency in cold primed plants under subsequent cold stress.

Photosynthetic electron transport generates ATP and NADPH to drive the photorespiratory C oxidation in the dark reaction in photosynthesis [39]. In chloroplast, Mg^2+^-ATPase and Ca^2+^-ATPase are important for the ATP formation [3]. In the present study, these two enzymes were both enhanced by the melatonin application during recovery of the cold primed plants. Together with melatonin’s positive effects on photosynthesis and antioxidant system, it could be concluded that applying melatonin during recovery enhanced cold priming that induced tolerance to subsequent cold stress in wheat.

## 4. Materials and Methods

### 4.1. Experimental Setup

**Figure 7:**
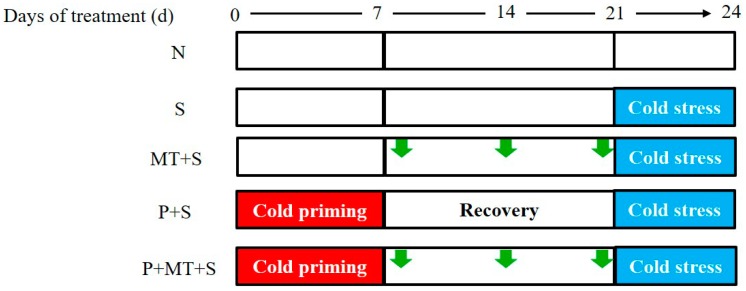
Schematic representation of experimental design and treatments. Abbreviations of treatments are explained in Figure 1.

The low-temperature-sensitive winter wheat cultivar Lianmai 6 was used for the experiment. Four seeds of winter wheat cv. Lianmai 6 were sown in each plastic pots (15 cm high and 25 cm in diameter), which was filled with 5 kg of clay soil. At the 3-leaf stage, only one seedling was retained after thinning. At the 4-leaf stage, the wheat plants were subjected to five treatments (Figure 7): N, the non-stress control, where wheat plants were grown at 26/18 °C (day/night); S, low temperature stress, in which the plants were exposed to a 3-day low temperature stress (2/0 °C in the day/night); P+S, cold priming + low temperature stress, where plants were first grown at 10/4 °C (day/night) for 7 days, then exposed to a 3-day low temperature stress (S) after a 14-day recovery; P+MT+S, cold priming + foliar melatonin application + low temperature stress, where the cold primed wheat plants were sprayed with 1mM melatonin three times during the recovery, then exposed to the low temperature stress; MT+S, foliar melatonin application + low temperature stress, where the non-primed plants were sprayed with 1mM melatonin and then exposed to the low temperature stress. The cold priming and low temperature stress treatment were applied in the temperature-control chamber. The photosynthetic active radiation was >500 μmol m^−2^ s^−1^ with a 12-hour photoperiod and the relative humidity was 70%. The experiment was a randomized block design, with three replicates for each treatment. Each replication consisted of 4 pots. For each replicate, one pot was used for gas exchange and membrane stability index measurement, one was used for antioxidant system analysis, and the rest of the two pots were used for qPCR and chloroplasts Isolation.

### 4.2. Gas Exchange and Chl a Fluorescence Transient

At the end of cold stress treatment, photosynthetic rate (*P_n_*) and stomatal conductance (*g_s_*) of the top leaf were measured with a portable photosynthesis system (LI-6400, LI-Cor, Lincoln, NE, USA) at a CO_2_ concentration of approximately 400 μmol mol^−1^ with a photosynthetically active radiation of 1200 μmol m^−2^ s^−1^. On each measurement, three leaves were taken for each treatment. Chlorophyll *a* fluorescence induction curve was measured with the same leaf as for the gas exchange analysis using Plant Efficiency Analyzer (Pocket-PEA, Hansatech, Norfolk, UK). Before measuring, 30 min of dark adaption of the leaves was needed. The collected data were processed by the program PEA Plus 1.04.

### 4.3. Membrane Stability Index

The measurement of membrane stability index (MSI) was applied with a conductivity meter. Leaf samples (200 mg) were washed in ddH_2_O and placed in two separate 10 mL tubes with ddH_2_O. One tube was heated at 40 °C for 30 min, while the other tube was boiled at 100 °C for 10 min. Subsequently, the electrical conductivity was measured for both (EC1 and EC2). The MSI was calculated by the following equation:

MSI = [1 − (EC1 − EC2)] × 100%(1)

### 4.4. H_2_O_2_ Concentration and Antioxidant Enzyme Activity

Following our previous methods, H_2_O_2_ concentration was measured by monitoring the absorbance of titanium peroxide complex at 410 nm [40]. APX activity was determined by monitoring the decrease at 290 nm, and the activity of SOD was measured by monitoring the inhibition of photochemical reduction of nitroblue tetrazolium (NBT) [12]. CAT activity was measured as described by Li et al. [13].

### 4.5. RNA Extraction and qRT-PCR

RNA was extracted from wheat leaves using Trizol according to the manufacturer’s instructions. The following primers were used for amplification as our previous experiment [10]: *Cu/Zn SOD*, 5′-CGCTCAGAGCCTCCTCTTT-3′ and 5′-CTCCTGGGGTGGAGACAAT-3′; *Fe SOD*, 5′-GAAGCTTGAGGTGGCACA-3′ and 5′-TAAGCATGCTCCCACAAGTC-3′; *CAT*, 5′-CCATGAGATCAAGGCCATCT-3′ and 5′-ATCTTACATGCTCGGCTTGG-3′; *tAPX*, 5′-GCAGCTGCTGAAGGAGAAGT-3′ and 5′-CACTGGGGCCACTCACTAAT-3′; *β-actin*, 5′-GCTCGACTCTGGTGATGGTG-3′ and 5′-AGCAAGGTCCAAACGAAGGA-3′. The qPCR analysis was performed using the TaKaRa^®^ SYBR Premix Ex Taq^TM^ II on an ABI PRISM 7300 Sequence Detection System (ABI, Foster, CA, USA) following our previous experiment. Each extraction and qRT-PCR was replicated three times. The quantification of mRNA levels was based on the relative quantification method (2^−ΔΔCt^).

### 4.6. Chloroplasts Isolation and ATPase Activity

Leaf samples (6 g) were ground in 30 mL of extraction buffer (0.45 M sucrose, 15 mM 3-(*N*-morpholino) propanesulfonic acid (MOPS), 1.5 mM ethylene glycol tetra acetic acid (EGTA), 0.6% polyvinylpyrro-lidone (PVP), 0.2% bovine serum albumine (BSA), 0.2 mM phenylmethylsulphonyl fluoride (PMSF) and 10 mM dithiothreitol (DTT)). Homogenate was filtered through gauze (8 layers), and the filtrate was centrifuged at 2000× *g* for 5 min. The sedimentation was resuspended with sorbitol resuspension medium (SRM, 0.33 M sorbitol in 50 mM 4-(2-hydroxyethyl)-1-piperazineethanesulfonic acid) and layered on the top of a layered system (7 mL, 35%, 80% Percoll) for the step gradients. The chloroplasts were collected and washed with 2 mL SRM followed by centrifugation at 1100× *g* for 10 min. The activities of Ca^2+^- and Mg^2+^-ATPase in the chloroplasts suspension were measured following the method proposed by Li et al. [3].

### 4.7. Statistical Analysis

The data was initially tested for homogeneity of variance with boxplot and subjected to *t*-test using SigmaSTAT (V3.5, Systat Software Inc., CA, USA).

## References

[1] Li X., Jiang H., Liu F., Cai J., Dai T., Cao W., Jiang D. (2013). Induction of chilling tolerance in wheat during germination by pre-soaking seed with nitric oxide and gibberellin. Plant Growth Regul..

[2] Shahryar N., Maali-Amiri R. (2016). Metabolic acclimation of tetraploid and hexaploid wheats by cold stress-induced carbohydrate accumulation. J. Plant Physiol..

[3] Li X., Hao C., Zhong J., Liu F., Cai J., Wang X., Zhou Q., Dai T., Cao W., Jiang D. (2015). Mechano-stimulated modifications in the chloroplast antioxidant system and proteome changes are associated with cold response in wheat. BMC Plant Biol..

[4] Baek K.-H., Skinner D.Z. (2003). Alteration of antioxidant enzyme gene expression during cold acclimation of near-isogenic wheat lines. Plant Sci..

[5] Gusta L.V., Wisniewski M. (2013). Understanding plant cold hardiness: An opinion. Physiol. Plant..

[6] Xu Q., Truong T.T., Barrero J.M., Jacobsen J.V., Hocart C.H., Gubler F. (2016). A role for jasmonates in the release of dormancy by cold stratification in wheat. J. Exp. Bot..

[7] Dahal K., Kane K., Sarhan F., Grodzinski B., Huner N.P.A. (2012). Cold acclimation inhibits CO_2_-dependent stimulation of photosynthesis in spring wheat and spring rye. Bot. Bot..

[8] Ruelland E., Vaultier M.-N., Zachowski A., Hurry V., Jean-Claude K., Michel D. (2009). Chapter 2 Cold Signalling and Cold Acclimation in Plants, in Advances in Botanical Research.

[9] Siddiqui K.S., Cavicchioli R. (2006). Cold-adapted enzymes. Annu. Rev. Biochem..

[10] Chinnusamy V., Zhu J., Zhu J.-K. (2007). Cold stress regulation of gene expression in plants. Trends Plant Sci..

[11] Winfield M.O., Lu C., Wilson I.D., Coghill J.A., Edward K.J. (2010). Plant responses to cold: Transcriptome analysis of wheat. Plant Biotechnol. J..

[12] Cevtkovic J., Muller K., Baier M. (2017). The effect of cold primng on the fitness of *Arabidopsis thaliana* accessions under natural and controlld conditios. Sci. Rep..

[13] Pomeroy M., Andrews C., Fedak G. (1975). Cold hardening and dehardening responses in winter wheat and winter barley. Can. J. Plant Sci..

[14] Li X., Cai J., Liu F., Dai T., Cao W., Jiang D. (2014). Cold priming drives the sub-cellular antioxidant systems to protect photosynthetic electron transport against subsequent low temperature stress in winter wheat. Plant Physiol. Biochem..

[15] Li X., Brestic M., Tan D.-X., Zivcak M., Zhu X., Liu S., Song F., Reiter R.J., Liu F. (2018). Melatonin alleviates low PS I limited carbon assimilation under elevated CO_2_ and enhances the cold tolerance of offspring in chlorophyll b-deficient mutant wheat. J. Pineal Res..

[16] Kobylińska A., Borek S., Posmyk M.M. (2018). Melatonin redirects carbohydrates metabolism during sugar starvation in plant cells. J. Pineal Res..

[17] Wang L., Feng C., Zheng X., Guo Y., Zhou F., Shan D., Liu X., Kong J. (2017). Plant mitochondria synthesize melatonin and enhance the tolerance of plants to drought stress. J. Pineal Res..

[18] Lee K., Lee H.Y., Back K. (2017). Rice histone deacetylase 10 and Arabidopsis histone deacetylase 14 genes encode N-acetylserotonin deacetylase, which catalyzes conversion of N-acetylserotonin into serotonin, a reverse reaction for melatonin biosynthesis in plants. J. Pineal Res..

[19] Lee K., Choi G.H., Back K. (2017). Cadmium-induced melatonin synthesis in rice requires light, hydrogen peroxide, and nitric oxide: Key regulatory roles for tryptophan decarboxylase and caffeic acid O-methyltransferase. J. Pineal Res..

[20] Lee K., Back K. (2017). Overexpression of rice serotonin N acetyltransferase 1 in transgenic rice plants confers resistance to cadmium and senescence and increases grain yield. J. Pineal Res..

[21] Li X., Wei J.-P., Scott E., Liu J.-W., Guo S., Li Y., Zhang L., Han W.-Y. (2018). Exogenous melatonin alleviates cold stress by promoting antioxidant defense and redox homeostasis in *Camellia sinensis* L.. Molecules.

[22] Han Q.-H., Huang B., Ding C.-B., Zhang Z.-W., Chen Y.-E., Hu C., Zhou L.-J., Huang Y., Liao J.-Q., Yuan S. (2017). Effects of melatonin on anti-oxidative systems and photosystem II in cold-stressed rice seedlings. Front. Plant Sci..

[23] Farquhar G.D., Sharkey T.D. (1982). Stomatal conductance and photosynthesis. Annu. Rev. Plant Physiol..

[24] Jarvis A.J., Mansfield T.A., Davies W.J. (1999). Stomatal behaviour, photosynthesis and transpiration under rising CO_2_. Plant Cell Environ..

[25] Sangwan V., Foulds I., Singh J., Dhindsa R.S. (2001). Cold-activation of Brassica napus BN115 promoter is mediated by structural changes in membranes and cytoskeleton, and requires Ca^2+^ influx. Plant J..

[26] Abid M., Ali S., Qi L.K., Zahoor R., Tian Z., Jiang D., Snider J.L., Dai T. (2018). Physiological and biochemical changes during drought and recovery periods at tillering and jointing stages in wheat (*Triticum aestivum* L.). Sci. Rep..

[27] Zhang X.-Y., Liang C., Wang G.-P., Luo Y., Wang W. (2010). The protection of wheat plasma membrane under cold stress by glycine betaine overproduction. Biol. Plant..

[28] Keunen E., Peshev D., Vangronsveld J., Van Den Ende W., Cuypers A. (2013). Plant sugars are crucial players in the oxidative challenge during abiotic stress: Extending the traditional concept. Plant Cell Environ..

[29] Asada K. (1999). The Water-water cycle in chloroplasts: Scavenging of active oxygens and dissipation of excess photons. Annu. Rev. Plant Physiol. Plant Mol. Biol..

[30] Li X., Tan D.X., Jiang D., Liu F. (2016). Melatonin enhances cold tolerance in drought-primed wild-type and abscisic acid-deficient mutant barley. J. Pineal Res..

[31] Brestic M., Zivcak M., Hauptvogel P., Misheva S., Kocheva K., Yang X., Li X., Allakhverdiev S.I. (2018). Wheat plant selection for high yields entailed improvement of leaf anatomical and biochemical traits including tolerance to non-optimal temperature conditions. Photosynth. Res..

[32] Zivcak M., Bruckova K., Sytar O., Brestic M., Olsovska K., Allakhverdiev S.I. (2017). Lettuce flavonoids screening and phenotyping by chlorophyll fluorescence excitation ratio. Planta.

[33] Brestic M., Zivcak M., Kunderlikova K., Allakhverdiev S.I. (2016). High temperature specifically affects the photoprotective responses of chlorophyll b-deficient wheat mutant lines. Photosynth. Res..

[34] Brestic M., Zivcak M., Kunderlikova K., Sytar O., Shao H., Kalaji H.M., Allakhverdiev S.I. (2015). Low PSI content limits the photoprotection of PSI and PSII in early growth stages of chlorophyll b-deficient wheat mutant lines. Photosynth. Res..

[35] Zivcak M., Kalaji H.M., Shao H.-B., Olsovska K., Brestic M. (2014). Photosynthetic proton and electron transport in wheat leaves under prolonged moderate drought stress. J. Photochem. Photobiol. B Biol..

[36] Zivcak M., Brestic M., Balatova Z., Drevenakova P., Olsovska K., Kalaji H.M., Yang X., Allakhverdiev S.I. (2013). Photosynthetic electron transport and specific photoprotective responses in wheat leaves under drought stress. Photosynth. Res..

[37] Spoustová P., Synková H., Valcke R., Čeřovská N. (2013). Chlorophyll a fluorescence as a tool for a study of the Potato virus Y effects on photosynthesis of nontransgenic and transgenic Pssu-ipt tobacco. Photosynthetica.

[38] Chen S., Yang J., Zhang M., Strasser R.J., Qiang S. (2016). Classification and characteristics of heat tolerance in Ageratina adenophora populations using fast chlorophyll a fluorescence rise O-J-I-P. Environ. Exp. Bot..

[39] Zuo Z., Sun L., Wang T., Miao P., Zhu X., Liu S., Song F., Mao H., Li X. (2017). Melatonin improves the photosynthetic carbon assimilation and antioxidant capacity in wheat exposed to nano-ZnO stress. Molecules.

[40] Li X., Cai J., Liu F., Dai T., Cao W., Jiang D. (2014). Exogenous abscisic acid application during grain filling in winter wheat improves cold tolerance of offspring’s seedlings. J. Agron. Crop Sci..

